# Time-for-space substitution in N-mixture models for estimating population trends: a simulation-based evaluation

**DOI:** 10.1038/s41598-021-84010-5

**Published:** 2021-02-25

**Authors:** Andrea Costa, Sebastiano Salvidio, Johannes Penner, Marco Basile

**Affiliations:** 1 Department of Earth and Life Sciences (DISTAV), University of Genova, Corso Europa 26, 16132 Genova, Italy; 2 Chair of Wildlife Ecology and Management, University of Freiburg, Tennenbacher Str. 4, 79106 Freiburg, Germany

**Keywords:** Conservation biology, Population dynamics, Biodiversity

## Abstract

N-mixture models usually rely on a meta-population design, in which repeated counts of individuals in multiple sampling locations are obtained over time. The time-for-space substitution (TSS) in N-mixture models allows to estimate population abundance and trend of a single population, without spatial replication. This application could be of great interest in ecological studies and conservation programs; however, its reliability has only been evaluated on a single case study. Here we perform a simulation-based evaluation of this particular application of N-mixture modelling. We generated count data, under 144 simulated scenarios, from a single population surveyed several times per year and subject to different dynamics. We compared simulated abundance and trend values with TSS estimates. TSS estimates are overall in good agreement with real abundance. Trend and abundance estimation is mainly affected by detection probability and population size. After evaluating the reliability of TSS, both against real world data, and simulations, we suggest that this particular application of N-mixture model could be reliable for monitoring abundance in single populations of rare or difficult to study species, in particular in cases of species with very narrow geographic ranges, or known only for few localities.

## Introduction

The study of population abundance and the analysis of its fluctuations over time, i.e. population dynamics, constitutes a fundamental tool in adaptive wildlife monitoring, conservation and management^1,2^. However, it is often difficult to obtain reliable estimates of population size, or even to accurately assess species presence, given that species within a community and individuals within a population are typically detected imperfectly^1,3^. In order to account for imperfect detection, researchers developed several methods for obtaining reliable estimates of abundance, such as capture-mark-recapture (CMR), removal and distance sampling^1,4^. These methods rely mostly on individual identification or some sort of auxiliary data (e.g. in distance sampling, the perpendicular distance between the observation and the transect) that may be expensive in terms of costs and efforts, impractical to collect or even unsuitable in complex environments or for small and secretive species. Over the last fifteen years the development and application of N-mixture models^5^, that allow simultaneous estimation of abundance and detection probability received large interest from field ecologists^6^. These methods rely on repeated counts of individuals at multiple sites (e.g. line transects or plots) and are highly cost-effective^7,8^. More recently, some extensions to these models have been formulated for various data types other than simple counts, such as removal or double observer data^9^, to model open populations in a robust-design fashion^10^, or even to model species-specific abundance of entire assemblages or communities^6,11^. Another important feature of N-mixture models is the capability of modelling both the observation process (i.e. detection probability) and the ecological processes (e.g. abundance) as a function of environmental covariates; hence allowing ecological inferences about abundance and density or to address management and conservation actions^12,13^. Increasing use of N-mixture modelling in monitoring schemes and ecological studies led to a growing interest in their reliability. Indeed, several studies raised issues with regard to parameter identifiability when model assumptions are violated or in presence of unmodelled sources of heterogeneity in the abundance or detection processes^14,15^. Nevertheless, despite the problems raised, many recent studies confirmed the reliability of this family of models in real-life applications: by comparing them against a hierarchical variant of a capture-recapture model^16^, or by comparing abundance estimates obtained by means of N-mixture models against those estimated with widely accepted methods, such as CMR, removal or distance sampling^7,17–20^. Although N-mixture modelling can be employed in many situations, this approach typically relies on a meta-population design, in which repeated counts of individuals in multiple sampling locations are obtained over time^6^. However, particularly when dealing with species with a very narrow geographic range and few known populations, a spatially replicated monitoring protocol may be impracticable, and only data for single populations in successive years may be available. In these situations, the application of time-for-space substitution (TSS) in the N-mixture modelling framework may be a suitable option to monitor a population on a single site over several years^20,21^. Time-for-space substitution is applied when multiple counts are conducted over regular time intervals (e.g. months, seasons or years) at the same site, and time replicates (i.e. years) are used in place of space replicates (i.e. sites), while within-year repeated counts (i.e. surveys) are employed as temporal replications, considering the population demographically closed within each year. This framework has been employed for a multi-species system with detection/non-detection data of bird species obtained during nine consecutive years at a single site in Japan^21^. It has also been applied on a 20-years spanning dataset of a declining population of an endangered gecko in Italy, finding good agreement with CMR estimates obtained from the same data, and hence validating the TSS framework on a real-life application^20^. Although an evaluation of this technique against a small simulation scenario already exists, the need of an in-depth evaluation through a more extensive simulation framework has been highlighted^6^.

Aim of this study is to provide an evaluation of the reliability of TSS applied to N-mixture models, and in particular to assess the reliability of abundance and trend estimations over several scenarios, including different population dynamics, initial abundance, detection probability, survey effort or duration of the study, by simulating count data from a single population surveyed several times per year and subject to different dynamics, and then comparing real abundance and trend values with TSS estimates.

## Methods

### Simulation scenarios

Binomial N-mixture models with TSS estimate latent abundance state *N* for year *i (N*_*i*_*),* assuming *N*_*i*_ ~ *Poisson*(*λ)*, where *λ* is the expected abundance over all years, by using repeated counts *C* at year *i* during survey *j* (*C*_*ij*_) to estimate individual detection probability *p*, assuming *C*_*ij*_*|N*_*i*_ ~ *Binomial(N*_*i*_*,p).* Both parameters can be modelled as a function of environmental covariates trough a log or logit link, respectively. In order to assess the reliability of the TSS in N-mixture model framework we built three main arrays of different scenarios, based on the underlying population dynamics (constant, positive or negative trend), each one containing 48 nested scenarios resulting from combinations of population initial abundance (N = 5, 20, 100; for small, medium and large populations, respectively), study duration (10, 20 years), survey effort (3, 5 surveys/year) and detection probability (p = 0.1, 0.3, 0.5, 0.7), thus obtaining 144 different scenarios. For each of these scenarios, we simulated 1000 data-sets obtaining a total of 144,000 data-sets. Simulated data were generated using the code from Kéry and Royle^6^ and a full version of our code is available as supplementary material (Appendix 1). Population dynamics are expressed as a log-linear regression coefficient^6^, formulated as

1 $$ log\left( {\lambda_{i} } \right) = \beta_{0} + \beta_{1} * \, Time_{i} $$

where *λ* is the abundance at year *i, β* are the model coefficients and *Time* is the year variable with length *i* equal to 10 or 20. We simulated *β*_*1*_ as a trend coefficient of T = 0.0 for the 48 constant population models, a coefficient of T = 0.1 for the 48 positive trend scenarios and a coefficient of T = -0.1 for the 48 models accounting for a declining population. Detection probability was expressed with a logit-linear regression coefficient, formulated as constant

2 $$ log\,it\left( {p_{ij} } \right) = \alpha_{0} $$

where *p* is the detection probability at year *i* and site survey *j*, which can have length equal to 3 or 5. Each dataset derived from our simulation process was analyzed by means of TSS N-mixture models. Our models accounted for Poisson error distribution and the upper boundary (K), i.e. the maximum value of integration of the abundance estimates, was automatically set for each model run; we did not consider negative binomial error distribution since it has been observed to cause infinite or K-truncated abundance estimates^14,22^. For each simulated dataset, we stored the real initial abundance (*N*) and the real trend (*T*; for those models including population dynamics), along with the estimated abundance ($\hat{N}$) and trend ($\hat{T}$), and then we calculated the relative deviation of each estimate from the true value of the corresponding parameter*.* While in the context of N-mixture model performance evaluation the term bias is usually employed to define the tendency of a simulation block for over- or under-estimation of a given parameter [e.g. 23–25], here we define it more widely as error. We calculated this relative deviation as follows: N-error = ($\hat{N}$—*N*) / *N* and T-error = ($\hat{T}$ – *T*) / *T*. For constant population models, it was not possible to calculate T-error, because of a division by zero, and therefore the distance of the observed trend from the real one was used. Thus, we obtained a relative measure of the error for each simulation, which will assume positive values in case of overestimation of the real parameter or will fall below zero in case of underestimation^23,24^. Moreover, this relative error can be interpreted as a percent measure: for instance, a N-error = 0.2 means that the given model overestimated abundance by 20%. Considering that a value of N-error < 0.20 (which equals a 20% over- or under-estimation for a given parameter) can still produce viable estimates for wildlife managing and monitoring programs^25^, we estimated the threshold values of detection probability needed to achieve this error in relation to population sizes, and independently from survey effort or study duration. We calculated the coverage probability, i.e. the proportion of simulations in which the true parameter is covered by the 95% Confidence Interval, for both abundance and trend estimates under a subset of scenarios, and evaluated if scenarios with a 95% or higher coverage rate showed lower error than those with a lower coverage. Finally, in order to evaluate the effect of unmodelled heterogeneity in abundance, on the performance of TSS, we calculated and retained a measure of heterogeneity for each dataset and compared it with N-error. As a measure of heterogeneity in abundance among years, we adopted the coefficient of variation (CV) of the real abundance of each year of the simulated study (Nhetereogeneity), calculated as the standard deviation of the real abundance of each year of the simulation scenario divided by the mean abundance of that scenario, following Duarte et al.^23^ and Costa et al.^24^. We performed this analysis only for the 48 models accounting for a constant population. All analyses were conducted in the R environment with packages “unmarked”^26^ and “AHMbook”^27^.

### Goodness-of-fit testing

Like any other estimator, also N-mixture models require a set of assumptions (demographic closure within a sampling season and independence of detection, among many –^5^) to be verified and met. The most adopted method to assess assumption violation in N-mixture models is to employ parametric bootstrapping procedure as a goodness-of-fit (GOF) test^6,28^. Thus, we performed a GOF test on a subset of our simulation scenarios to evaluate the reliability of parametric bootstrap GOF test in identifying problems and predicting deviation from real parameters’ values in N-mixture model estimates with TSS, following Duarte et al.^23^ and Knape et al.^29^. We simulated again our 48 constant population scenarios, but this time we also performed a Pearson chi-square test using a parametric bootstrap procedure with 1000 resampling, for each fitted model^28^. We considered models to result in a good fit when the p-value of the Pearson chi-square was > 0.1. Moreover, for each fitted model, we also obtained a measure of overdispersion (c-hat) which assumes values close to 1.0 for good fitting models, and increase (or decrease towards 0) in case of assumption violations. Then, we compared N-error of each model with its corresponding GOF test results, highlighting whether GOF test is a good predictor of model error, i.e. identifying models with higher under- or over-estimation of abundance. Since performing parametric bootstrap GOF test is time consuming, for this analysis we reduced the number of simulations for each of the 48 scenarios from 1000 to 200. GOF tests were performed in R environment with package “AICcmodavg”^30^.

## Results

### Simulation scenarios

**Figure 1:**
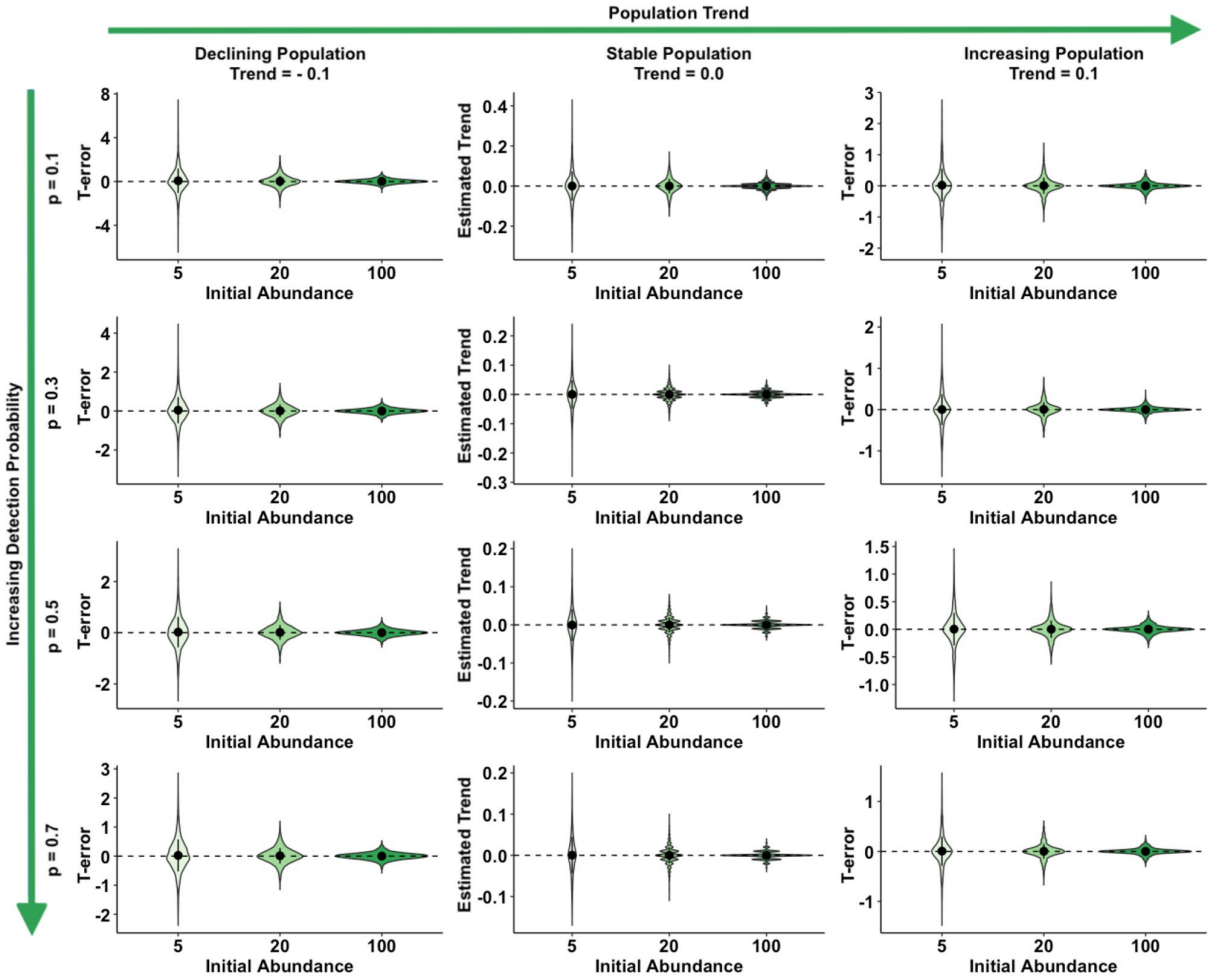
Violin plots of the observed error in trend estimation (T-error) for several scenarios, according to different levels of population trend, detection probability, and initial abundance. Black dots and vertical bars inside each violin plot represent the mean and standard deviation, respectively.

**Figure 2:**
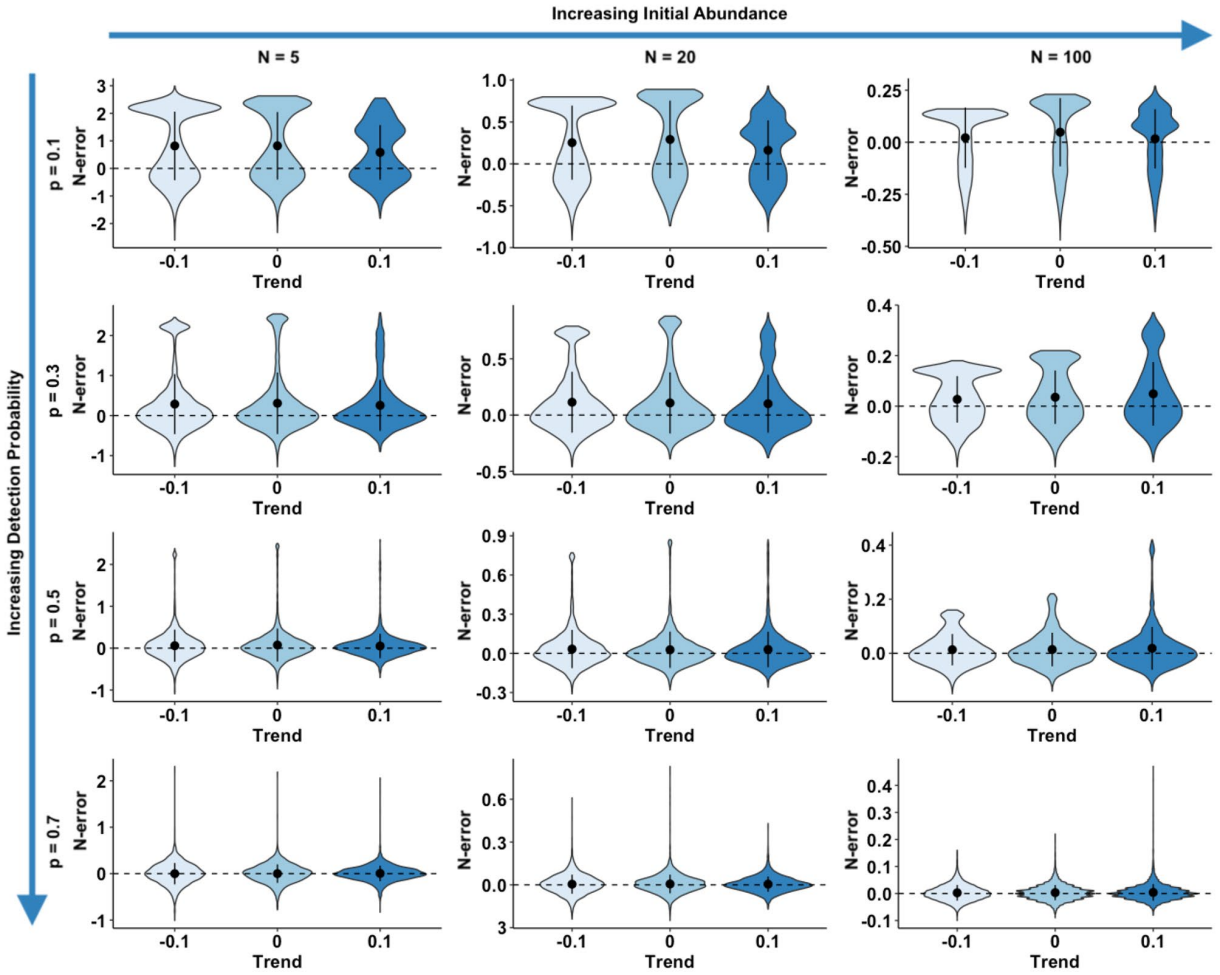
Violin plots of the observed error in abundance estimation (N-error) for several scenarios, according to different levels of initial abundance, detection probability, and population trend. Black dots and vertical bars inside each violin plot represent the mean and standard deviation, respectively.

**Figure 3:**
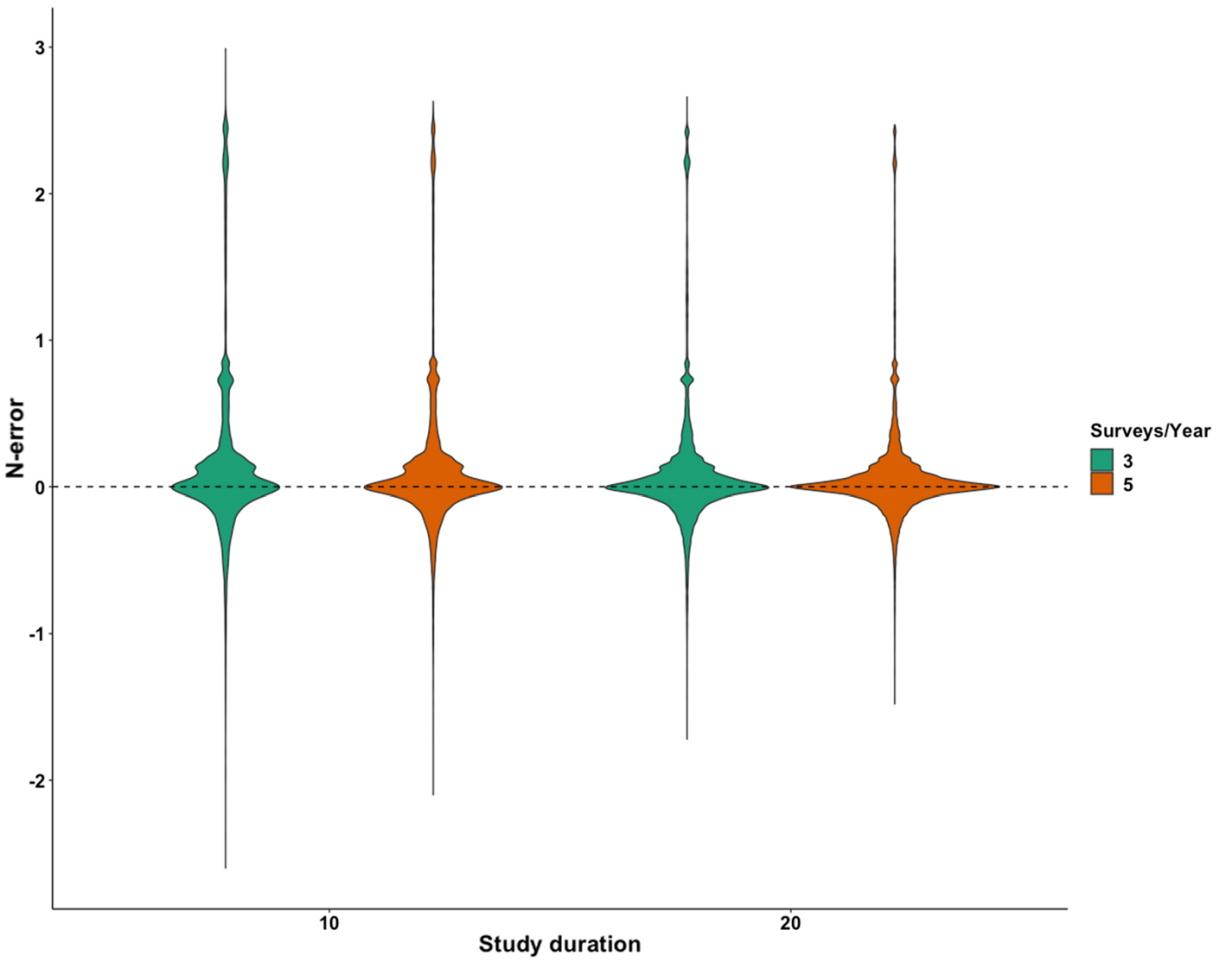
Violin plots of the observed error in abundance estimation (N-error) for different levels of study duration (10 and 20 years) and survey effort (3 and 5 surveys per year).

**Figure 4:**
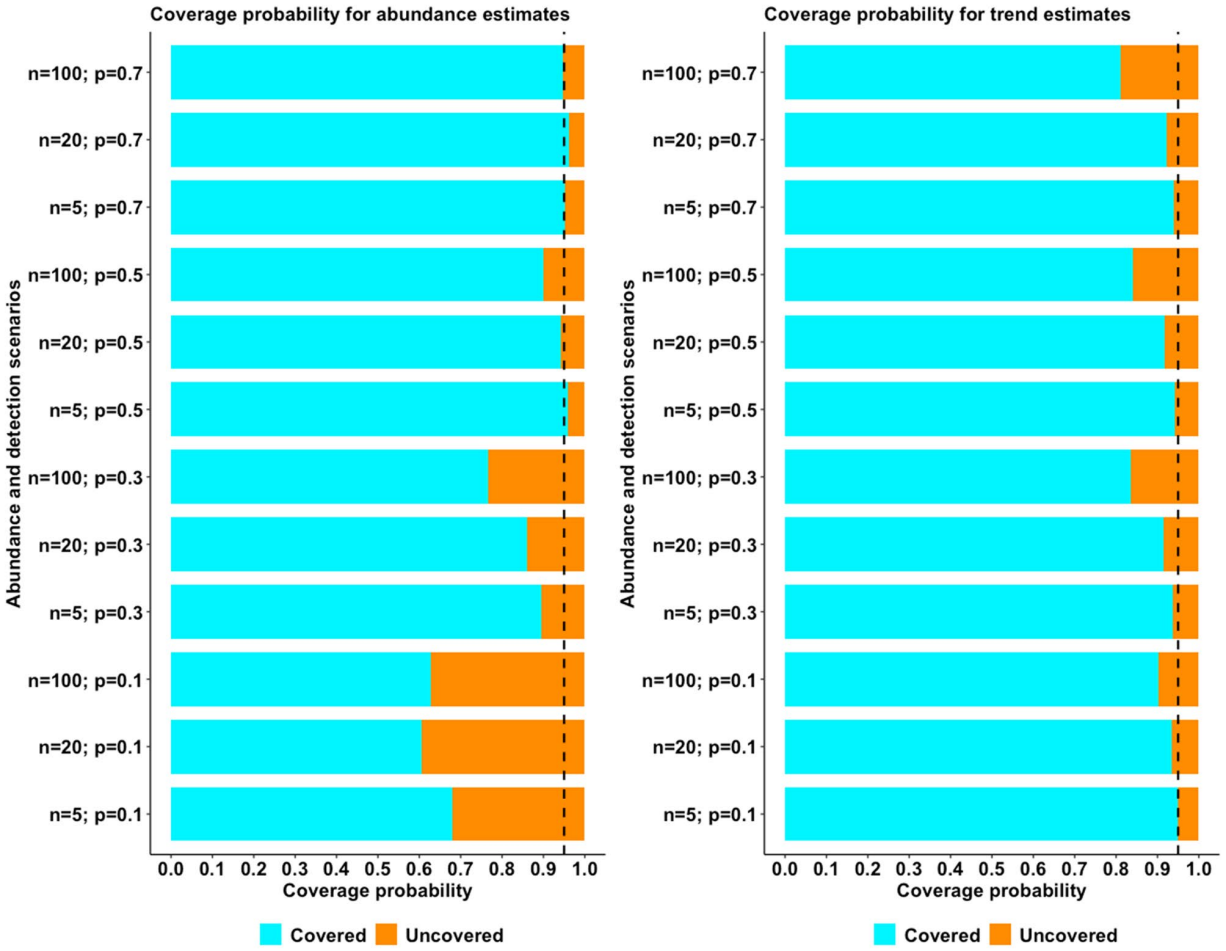
Bar charts representing the proportion of models in which real parameters are covered by the 95% confidence interval. The vertical dashed line represents a 0.95 coverage probability.

**Figure 5:**
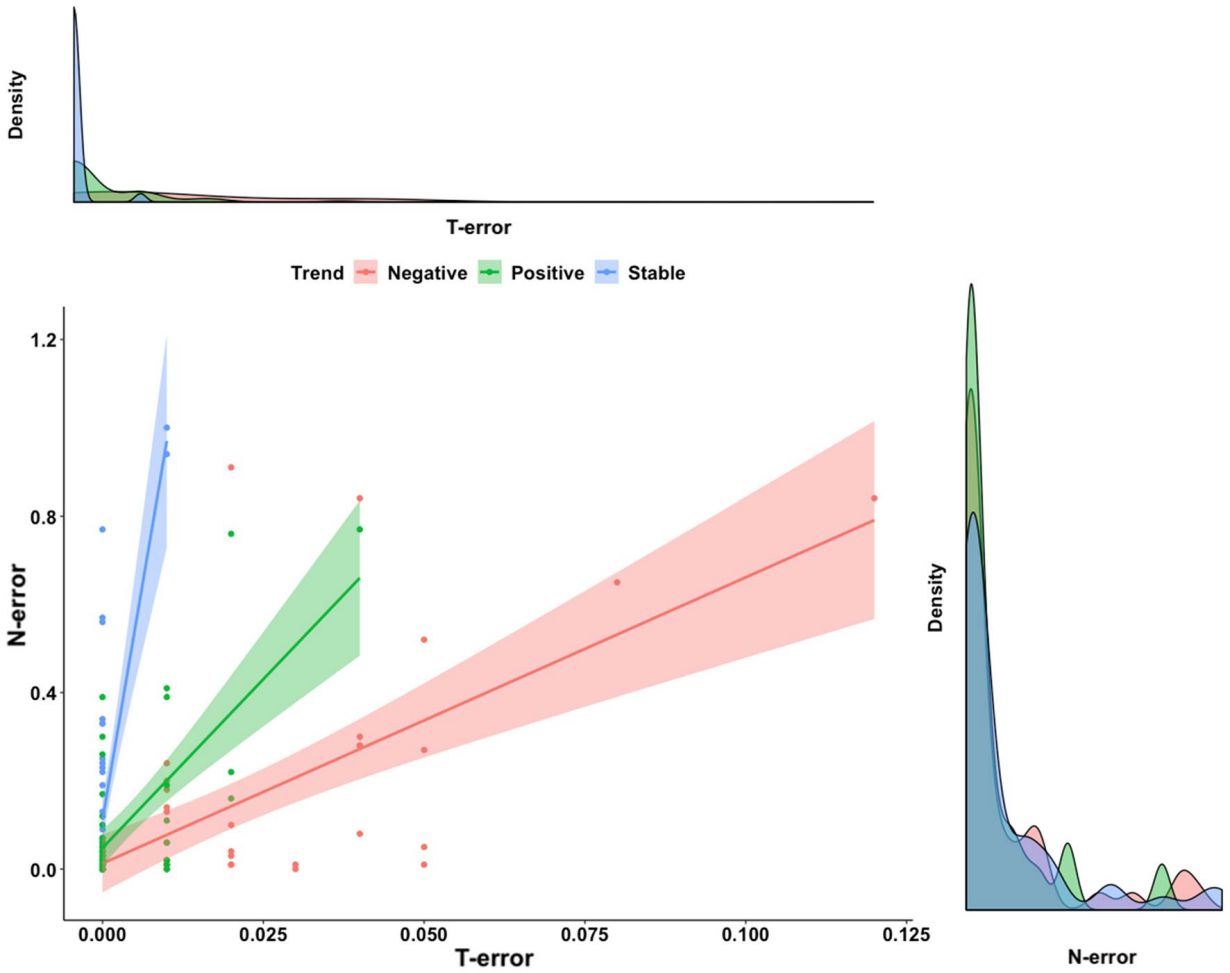
Scatterplots representing the relationship between the absolute value of error in abundance estimation and the error in trend estimation, for the 144 simulation scenarios. The density plot on the top and the one on the right represent the distribution of the error in trend estimation and abundance, respectively.

**Figure 6:**
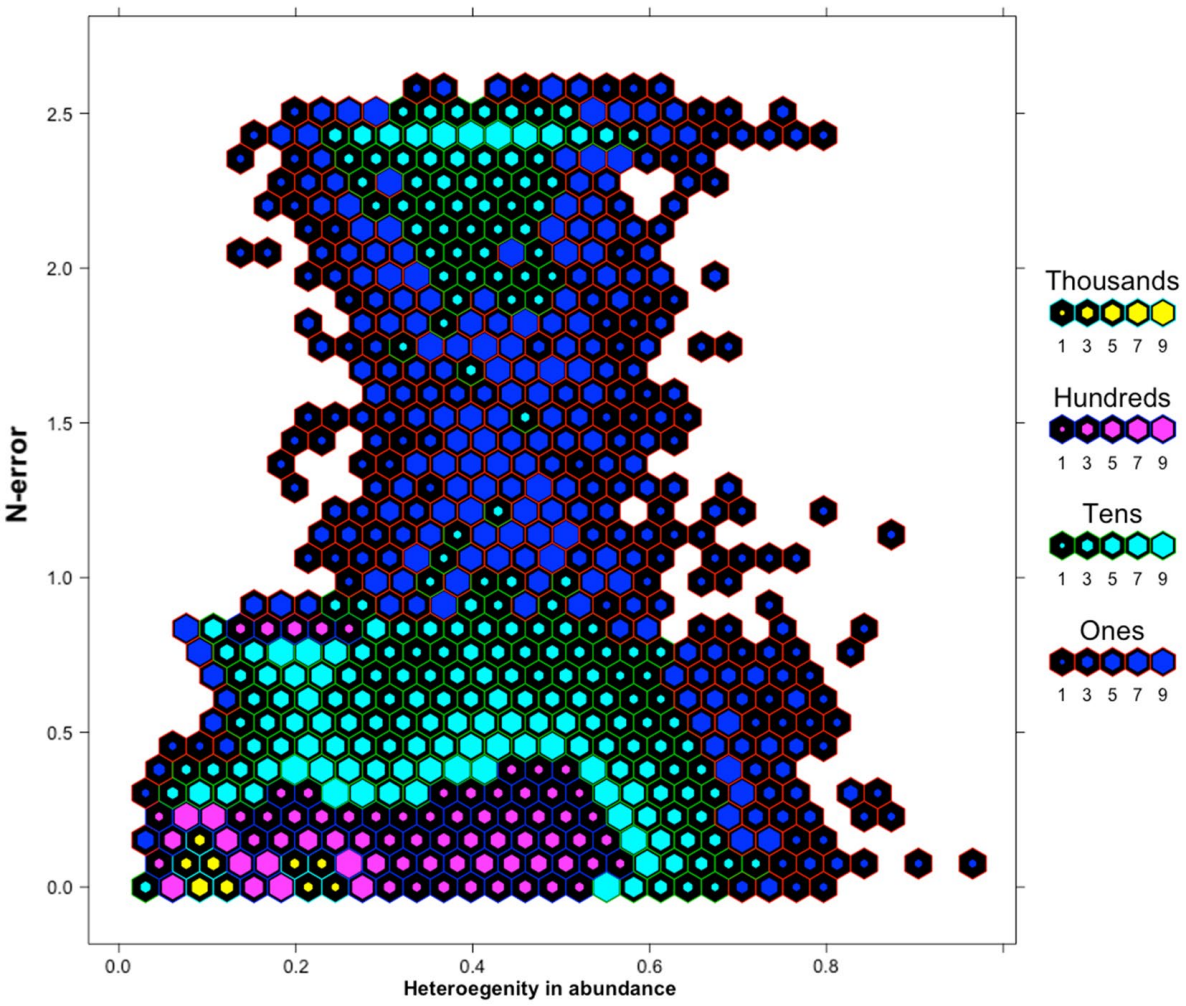
Scatterplot of the relationship between heterogeneity in abundance and the absolute value of error in abundance estimation for stable population scenarios. Hexagonal bins represent the number of points falling within each hexagon.

A graphic summary of results from the simulation scenarios, in trend and abundance estimation, is presented in Figs. 1 and 2, respectively, while complete results are reported as supplementary materials (Supplementary Tables 1 and 2). With regard to trend estimation, error in estimates was generally higher in declining rather than in stable or increasing populations (Fig. 1). Similarly, simulation scenarios accounting for a lower initial abundance (N = 5) experienced the higher T-error while the lower T-error value was achieved in larger populations (Fig. 1). Detection probability seemed to have a great influence on trend estimation: the scenarios accounting for a lower detection probability (p = 0.10 and 0.30) being the ones with higher T-error and, conversely, the ones with higher detectability (p = 0.50 and 0.70) achieving the lower values of T-error*.* N-mixture models with TSS, within our range of scenarios, seemed to systematically overestimate abundance, being the value of N-error positive in the majority of cases. As already observed for T-error, also N-error was higher in populations experiencing a negative trend, rather than population with a positive or constant dynamic (Fig. 2). Moreover, error in abundance was sligthly influenced by initial population size, duration of the study and survey effort (Fig. 3). Finally, with regards to detection probability, the majority of simulations in small population scenarios achieved an N-error < 0.20 for detection probability p ≥ 0.5. In medium sized population scenarios, the threshold value of detection probability required to achieve an N-error < 0.20 decreases to p ≥ 0.3; while in case of large populations, even the majority of scenarios accounting for a p ≥ 0.1 show an N-error < 0.20. With regard to coverage rate of the 95% confidence interval in abundance and trend estimation, the results are summarized in Fig. 4. Scenarios characterized by lower detection probability were those experiencing the lower coverage probability for abundance estimates, while the coverage rate for trend estimates was higher also for these scenarios. The mean N-error of scenarios with a 95% coverage of the real abundance (mean = 0.05; sd = 0.33) was significantly lower than the mean N-error of uncovered scenarios (mean = 0.54; sd = 0.88; Mann–Whitney test, p < 0.001). Also for the deviation in trend estimation, the mean error was lower in scenarios with a coverage rate higher than 0.95 (mean = 0.004; sd = 0.56) than in those with a lower coverage rate (mean = 0.008; sd = 0.28; Mann–Whitney test, p < 0.001). For what concerns the relationship between N-error and T-error: even when a severe deviation in initial abundance is present, the error in trend estimation remained low, as shown in Fig. 5. The observed heterogeneity in abundance (Nhetereogeneity) in the 48 stable population scenarios ranged from 0.03 to 0.95 (mean = 0.25; sd = 0.15). The relationship between heterogeneity in abundance and N-error is shown in Fig. 6, and the correlation between them was weak but highly significant (Spearman’s rho = 0.39; p < 0.001).

### Goodness-of-fit testing

**Figure 7:**
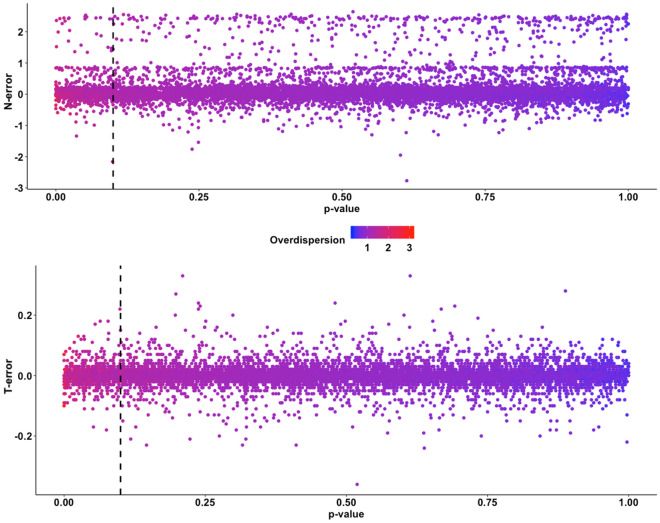
Scatterplots representing the relationship between the significance of the goodness of fit test and the error in abundance (top) and trend (bottom) estimation. Vertical dashed line represents the significance threshold of the test.

Results on goodness-of-fit test evaluation on our subset of 48 stable population scenarios are summarized in Fig. 7. Overall, the outcome of parametric bootstrap GOF test seemed unrelated with the error of abundance estimates. The majority of models evaluated in this simulation passed the threshold p-value (p = 0.10) for the GOF test, hence resulting in a theoretical good fit. However, some level of error was experienced by both models that resulted in a good fit and those that did not pass the GOF test. As shown in Fig. 7, a considerable number of models showed a high value of N-error and T-error, while their GOF p-value > 0.10. At the same time the value of c-hat did not relate with error. Both regularly over- and under-dispersed models experienced a large range of deviation from real values of abundance and trend.

## Discussion

From our simulation study, we observed how trend and abundance estimation is mainly affected by detection probability and population size. Error in trend estimation was also affected by trend direction: being higher in declining populations and particularly in case of low detection probability. In these cases, counts became small and might generate unreliable abundance estimates^5,14,31,32^. This pattern in trend estimation has also been observed for the open population N-mixture model^10,25^. At the same time, however, Ficetola et al.^25^ reported that error in abundance estimation, for the Dail Madsen model, is dependent on detection probability and survey effort, as in the present study, but not on initial population size. In the only evaluation of TSS against real world data Costa et al.^20^ observed how TSS may under-estimates abundance; an issue that is known to occur in Poisson N-mixture models in case of intrinsic heterogeneity, due to positive density-dependent detection^32^. Conversely, in the present study TSS tended to systematically over-estimate abundance: this behavior being known in case of low detection probability or in presence of unmodeled heterogeneity in the detection process^23^. Considering only detection probability and population size, we observed that at least a detection probability of p = 0.5 is required to decrease error in abundance estimate to < 20% for small populations, as already suggested for the classical N-mixture model^32^. In case of larger populations reliable estimates can be obtained for detectability ≥ 0.3 or even ≥ 0.1. Ecologists and wildlife managers usually need to estimate abundance and trend of small populations of rare, cryptic species: in these specific situations, it seems that TSS could not produce reliable estimates of abundance. However, we stress that our scenario for small populations was really severe. Indeed, in real world application ecologists and managers rarely deal with populations where N = 5, but is rather more likely to deal with larger ones^18,33^. In these cases, an estimation error lower than 20% is achievable despite low detection probability. Furthermore, results of our study showed that, even if a large error in abundance estimate may occur, associated estimates of population trend were still reliable. One of the major sources of criticism against N-mixture models concerns their lack of robustness in presence of, even slight, assumption violations. Main assumptions of N-mixture models are population closure, independence of detections, absence of individual heterogeneity in detection probability and, lastly, absence of unmodelled heterogeneity in abundance and detection^6^. Among these deviations from model assumptions, those regarding population closure and intrinsic heterogeneity cannot be addressed in N-mixture models with TSS, because the data do not retain individual identity. These latter sources of heterogeneity are those most investigated by recent studies on the effectiveness of N-mixture models^15,23,32^. Unmodelled heterogeneity in abundance and detection, by contrast, can be easily addressed by the use of covariates in N-mixture models, but its effect on model performance has not been tested in depth^6^. We tested this latter type of heterogeneity in abundance, across sampling years, in the TSS framework. The levels of heterogeneity in abundance in our simulated scenarios were slightly higher than those used in other studies^23^, and similar to those observed, for instance, in long-term studies of salamanders (CV = 0.27;^34^) and geckos (CV = 0.37;^20^) populations. The relationship between N-error and Nheterogeneity was weak and its effect seemed negligible. Moreover, this kind of unmodelled heterogeneity is easily addressable by the inclusion of covariates in the model^6^. In any case, we suggest that this topic should be analyzed in more detail in further studies. N-mixture models in general, like any other method for abundance estimation, require a set of assumptions to be met prior to fitting the model^5,6,28^. Methods to assess assumption violation in N-mixture models, and hierarchical models in general, are receiving increasing attention and their reliability as good diagnostic tools to assess model adequacy is currently under debate^23,29,35^. However, a proper goodness-of-fit test or an evaluation of model adequacy in N-mixture models is often overlooked^36,37^: even when evaluating their reliability relative to other methods^19,31^. In our study, the parametric bootstrap GOF test seemed to be somewhat unreliable for assessing the fit of TSS. Indeed, both the significance level of the test and its measure of overdispersion were unrelated to the deviation of the model from real values. However, Duarte et al.^23^, assessing the reliability of the parametric bootstrap GOF test for N-mixture models, found that the test adequately predicts estimation error when assumptions are met and detection probability is high, but with low detection probability the amount of false negative results (i.e. GOF test is not significant but deviation of estimates from real parameter values is high) increases and the model diagnostic becomes unreliable. From our screening of the parametric bootstrap GOF, it seemed that in TSS framework this model diagnostic is even more unreliable. This issue was probably due to the small sample size used in our simulation scenarios (i.e. number of years in TSS or sites in N-mixture model with meta-population design). Even if parametric bootstrap GOF test seems to be unreliable for testing model adequacy, other techniques and GOF checks have been proposed and found to be more reliable in evaluating model fit, such as quasi-coefficient of variation^23^ or analysis of residuals^29^.

Our study represents the first and until now the most complete evaluation of TSS for long-term population monitoring. After evaluating the reliability of TSS, both against real world data^20^ and against simulations, we suggest that this particular application of N-mixture model could be reliable for monitoring abundance and estimate trend in single populations, over several years. The poor reliability of the estimates in low detection probability situations, together with small population size, is in our opinion the major flaw of the method. However, a cost-effective and reliable technique allowing abundance or density estimation in these conditions is still lacking, but strongly needed. In more favorable conditions (i.e. p ≥ 0.3 or N ≥ 20) TSS showed a low error both in trend and abundance estimation and then we suggest that it should be considered as a good alternative for long term monitoring, after a period of validation with other methods^2^. Our findings are also remarkable in the light of local wildlife regulations or supranational directives, and in particular in the case of the Council of Europe Directive 92/43/EEC, known as Habitat Directive. In fact, article 11 of this Directive requires the application of reliable monitoring protocols in order to estimate the conservation status of dozens of animal and plant species listed in Annex IV. The Directive also imposes to Member States the maintenance of a “favorable” conservation status of the protected species, on the basis of population dynamics and natural range data. The long-term effort needed to obtain a reliable sample size may narrow down the application of TSS to few real-world cases. However, threatened species have often very narrow geographic ranges, or sometimes are known only from few local populations, that can be considered demographically closed for a short survey period each year. In these situations, the application of time-consuming protocols, such as CMR, removal or distance sampling, to the species long-term monitoring seems unrealistic and the conservation and management of these species may benefit from the application of more cost-effective monitoring methods, based on repeated count of unmarked individuals, such as TSS. Further developments of the model may be directed toward the relaxation of the closure assumption and the inclusion of temporal dependence structure among years.

## Supplementary Information

Supplementary Information 1.

Supplementary Information 2.
