# Supplementary material for: Time-for-space substitution in N-mixture models for estimating population trends: a simulation-based evaluation
Source: Sci Rep. 2021 Feb 25;11:4581. doi: 10.1038/s41598-021-84010-5 (PMC7907346; doi:10.1038/s41598-021-84010-5)
Supplement: Supplementary file 1 — Supplementary Information 1. [file 41598_2021_84010_MOESM1_ESM.docx]

Time-for-space substitution in N-mixture models for estimating population trends: a simulation-based evaluation

Andrea Costa^1^, Sebastiano Salvidio^1^, Johannes Penner^2^, Marco Basile*^2^

^1^ University of Genova - Department of Earth and Life Sciences (DISTAV), Corso Europa 26, 16132, Genova, Italy

^2^ Chair of Wildlife Ecology and Management, University of Freiburg, Tennenbacher Str. 4, D-79106, Freiburg, Germany

*Corresponding Author: Marco Basile – [marco.basile@wildlife.uni-freiburg.de](mailto:marco.basile@wildlife.uni-freiburg.de) ORCID: 0000-0003-0237-5482

Supplementary Material 1: code.

#Example code for simulating data, analyse counts and store results

#Complete analysis for 144 simulated scenarios and gof testing of 48 scenarios took approx. 14 daays of computing on a PC with Intel I7 quad-core SkyLake processor (3.6 GHz) and 16 GB RAM.

#Packages needed for simulating data, analyse counts and perform GOF test

library("AHMbook")

library("AICcmodavg")

library("unmarked")

#dataset created with function simpleNmix (package AHMbook)

#nyear -> Number of primary sampling periods.

#nrep -> Number of secondary samples within each primary period.

#beta0 -> the intercept of a log-linear model of expected abundance (lambda).

#beta1 -> the Time coefficient of a log-linear model for lambda (trend).

#alpha0 -> the intercept of a logit-linear model for detection (p).

#alpha1 -> the Time coefficient of a logit-linear model for detection (discarded in these simulations).

#alpha2 -> the coefficient of a survey-specific covariate such as temperature (discarded in these simulations).

#show.plot -> choose whether to show plots or not

#parameters used for designing scenarios

#n years= 10;20

#n survey/year (survey effort)=3;5

#N (population inital abundance, in log scale)=5;20;100

#trend (included as a loglinear covariate on abundance)=0.0; 0.1;-0.1

#p (detection probability, in logit scale)=0.1;0.3;0.5;0.7

#Example of a positive trend scenario

#Positive-trend-scenario 1

#n years=10

#n survey/year=3

#N=5

#trend=0.0

#p=0.1

simrep <- 1000 # Number of simreps

resultsPT1 <- array(NA, dim = c(simrep, 17)) # Array for results storage

for(i in 1:simrep){

cat("Simrep", i, "\n")

data<-simpleNmix(nyear = 10, nrep = 3, beta0 = 1.61, beta1 = 0.1, alpha0 = -2.197, alpha1 = 0,

alpha2 = 0, show.plot = FALSE) # Simulate a data set

umf <- unmarkedFramePCount(y = data$C, siteCovs = data.frame(Time = data$Time),

obsCov = NULL) #Create Unmarked frame object

Kset<-(max(data$C))*10+300 #Automatically set the upper integration limit, as a function of maximum count

tryCatch(fm1 <- pcount(~1 ~Time, data = umf,K=Kset, mixture="P"),error=function(e){}) #model run, nested within a error suppressing and storing function (tryCatch)

CI<-confint(fm1, type="state", level = 0.95) #Confidence intervals

resultsPT1[i, 1:3] <- round(coef(fm1),2) #Initial abundance, trend and detection estimates

resultsPT1[i, 4] <- round(qlogis(data$p[1,1]),2) #Real detection parameter

resultsPT1[i, 5] <- round(data$beta0,2) #Real abundance parameter

resultsPT1[i, 6] <- round(data$beta1,2) #Real trend parameter

resultsPT1[i, 7] <-round(((coef(fm1)[2])-(data$beta1))/(data$beta1),2) #Tbias

resultsPT1[i, 8] <-round(((coef(fm1)[1])-(data$beta0))/(data$beta0),2) #Nbias

resultsPT1[i, 9] <-round((sd(data$N))/(mean(data$N)),2) #Nheterogeneity (CV)

resultsPT1[i, 10] <-round(CI[1,1],2) #Lower limit of 95% Confidence Interval of abundance estimate

resultsPT1[i, 11] <-round(CI[1,2],2) #Upper limit of 95% Confidence Interval of abundance estimate

resultsPT1[i, 12] <-round(CI[2,1],2) #Lower limit of 95% Confidence Interval of trend estimate

resultsPT1[i, 13] <-round(CI[2,2],2) #Upper limit of 95% Confidence Interval of trend estimate

resultsPT1[i, 14] <-findInterval(round(data$beta0,2), c((round(CI[1,1],2)),(round(CI[1,2],2)))) #Coverage of real parameter for abundance

resultsPT1[i, 15] <-findInterval(round(data$beta1,2), c((round(CI[2,1],2)),(round(CI[2,2],2)))) #Coverage of real parameter for trend

resultsPT1[i, 16] <-data$nyear #Study duration

resultsPT1[i, 17] <-data$nrep #Sampling effort

}

colnames(resultsPT1) <- c("lam(Int)","lam(Time)","p(Int)","Preal","Nreal","Treal","Terror","Nerror","Nheterogeneity",

"lowN","upN","lowT","upT","CoverageN","CoverageT","Nyear","Nrep") #column names

#Example of a stable population scenario with GOF test

#similar to the previous example but includes code for "Nmix.gof.test" function in package "AICcmodavg"

#NO-trend-scenario 1

#n years=10

#n survey/year=3

#N=5

#trend=0.0

#p=0.1

simrep <- 200

resultsGOF1 <- array(NA, dim = c(simrep, 20))

for(i in 1:simrep){

cat("Simrep", i, "\n")

data<-simpleNmix(nyear = 10, nrep = 3, beta0 = 1.61, beta1 = 0.0000001, alpha0 = -2.197, alpha1 = 0,

alpha2 = 0, show.plot = FALSE)

umf <- unmarkedFramePCount(y = data$C, siteCovs = data.frame(Time = data$Time),

obsCov = NULL)

tryCatch(fm1 <- pcount(~1 ~Time, data = umf,K=300, mixture="P"),error=function(e){})

CI<-confint(fm1, type="state", level = 0.95)

tryCatch(gof<- Nmix.gof.test(fm1, nsim = 1000, plot.hist = FALSE, report = NULL),error=function(e){}) #model run, nested within a error suppressing and storing function (tryCatch)

resultsGOF1[i, 1:3] <- round(coef(fm1),2)

resultsGOF1[i, 4] <- round(qlogis(data$p[1,1]),2)

resultsGOF1[i, 5] <- round(data$beta0,2)

resultsGOF1[i, 6] <- round(data$beta1,2)

resultsGOF1[i, 7] <-round(((coef(fm1)[2])-(data$beta1)),2)

resultsGOF1[i, 8] <-round(((coef(fm1)[1])-(data$beta0))/(data$beta0),2)

resultsGOF1[i, 9] <-round((sd(data$N))/(mean(data$N)),2)

resultsGOF1[i, 10] <-round(CI[1,1],2)

resultsGOF1[i, 11] <-round(CI[1,2],2)

resultsGOF1[i, 12] <-round(CI[2,1],2)

resultsGOF1[i, 13] <-round(CI[2,2],2)

resultsGOF1[i, 14] <-findInterval(round(data$beta0,2), c((round(CI[1,1],2)),(round(CI[1,2],2))))

resultsGOF1[i, 15] <-findInterval(round(data$beta1,2), c((round(CI[2,1],2)),(round(CI[2,2],2))))

resultsGOF1[i, 16] <-data$nyear

resultsGOF1[i, 17] <-data$nrep

resultsGOF1[i, 18] <-gof$p.value

resultsGOF1[i, 29] <-round(gof$c.hat.est,2)

}

colnames(resultsGOF1) <- c("lam(Int)","lam(Time)","p(Int)","Preal","Nreal","Treal","Terror","Nerror","Nheterogeneity",

"lowN","upN","lowT","upT","CoverageN","CoverageT","Nyear","Nrep","gof.p","gof.chat")
