# Supplementary material for: Time-for-space substitution in N-mixture models for estimating population trends: a simulation-based evaluation
Source: Sci Rep. 2021 Feb 25;11:4581. doi: 10.1038/s41598-021-84010-5 (PMC7907346; doi:10.1038/s41598-021-84010-5)
Supplement: Supplementary file 2 — Supplementary Information 2. [file 41598_2021_84010_MOESM2_ESM.docx]

Time-for-space substitution in N-mixture models for estimating population trends: a simulation-based evaluation

Andrea Costa^1^, Sebastiano Salvidio^1^, Johannes Penner^2^, Marco Basile*^2^

^1^ University of Genova - Department of Earth and Life Sciences (DISTAV), Corso Europa 26, 16132, Genova, Italy

^2^ Chair of Wildlife Ecology and Management, University of Freiburg, Tennenbacher Str. 4, D-79106, Freiburg, Germany

*Corresponding Author: Marco Basile – [marco.basile@wildlife.uni-freiburg.de](mailto:marco.basile@wildlife.uni-freiburg.de) ORCID: 0000-0003-0237-5482

Supplementary material 2: tables.

Table 1. Mean and standard deviation of N-error for 144 simulated scenarios

|  |  |  | **Negative Trend** | | |  |  |  | **Stable** | |  |  |  |  | **Positive Trend** | | |  |  |  |
| --- | --- | --- | --- | --- | --- | --- | --- | --- | --- | --- | --- | --- | --- | --- | --- | --- | --- | --- | --- | --- |
|  |  |  | **N = 5** |  | **N = 20** | | **N = 100** | | **N = 5** |  | **N = 20** | | **N = 100** | | **N = 5** |  | **N = 20** | | **N = 100** | |
|  |  |  | Mean | Sd | Mean | Sd | Mean | Sd | Mean | Sd | Mean | Sd | Mean | Sd | Mean | Sd | Mean | Sd | Mean | Sd |
| **10 Years** | **3 Surveys / Year** | p = 0.1 | 0,91 | 1,51 | 0,28 | 0,49 | 0,01 | 0,17 | 1,00 | 1,34 | 0,33 | 0,51 | 0,05 | 0,19 | 0,76 | 1,22 | 0,22 | 0,44 | 0,02 | 0,18 |
|  |  | p = 0.3 | 0,52 | 1,00 | 0,18 | 0,34 | 0,03 | 0,11 | 0,56 | 1,02 | 0,22 | 0,38 | 0,04 | 0,13 | 0,39 | 0,84 | 0,16 | 0,33 | 0,07 | 0,17 |
|  |  | p = 0.5 | 0,14 | 0,56 | 0,06 | 0,21 | 0,02 | 0,07 | 0,19 | 0,59 | 0,06 | 0,22 | 0,02 | 0,08 | 0,17 | 0,52 | 0,06 | 0,20 | 0,03 | 0,11 |
|  |  | p = 0,7 | 0,01 | 0,29 | 0,01 | 0,08 | 0,01 | 0,04 | 0,02 | 0,24 | 0,01 | 0,10 | 0,00 | 0,04 | 0,02 | 0,24 | 0,01 | 0,07 | 0,01 | 0,05 |
|  | **5 Surveys / Year** | p = 0.1 | 0,84 | 1,25 | 0,30 | 0,43 | 0,03 | 0,14 | 0,94 | 1,24 | 0,34 | 0,47 | 0,06 | 0,16 | 0,77 | 1,07 | 0,26 | 0,38 | 0,06 | 0,15 |
|  |  | p = 0.3 | 0,28 | 0,72 | 0,13 | 0,28 | 0,03 | 0,09 | 0,33 | 0,79 | 0,13 | 0,29 | 0,04 | 0,11 | 0,30 | 0,67 | 0,12 | 0,28 | 0,07 | 0,14 |
|  |  | p = 0.5 | 0,05 | 0,37 | 0,04 | 0,15 | 0,01 | 0,06 | 0,05 | 0,36 | 0,02 | 0,13 | 0,02 | 0,06 | 0,05 | 0,31 | 0,03 | 0,13 | 0,02 | 0,07 |
|  |  | p = 0,7 | -0,01 | 0,25 | 0,00 | 0,07 | 0,00 | 0,03 | 0,00 | 0,20 | 0,00 | 0,06 | 0,00 | 0,03 | 0,00 | 0,17 | 0,00 | 0,06 | 0,00 | 0,03 |
| **20 Years** | **3 Surveys / Year** | p = 0.1 | 0,84 | 1,26 | 0,24 | 0,45 | 0,02 | 0,15 | 0,77 | 1,23 | 0,25 | 0,46 | 0,04 | 0,16 | 0,41 | 0,82 | 0,07 | 0,29 | -0,02 | 0,12 |
|  |  | p = 0.3 | 0,27 | 0,70 | 0,10 | 0,26 | 0,02 | 0,09 | 0,24 | 0,67 | 0,09 | 0,26 | 0,03 | 0,10 | 0,19 | 0,51 | 0,07 | 0,21 | 0,04 | 0,12 |
|  |  | p = 0.5 | 0,03 | 0,31 | 0,02 | 0,11 | 0,01 | 0,05 | 0,06 | 0,31 | 0,02 | 0,11 | 0,01 | 0,06 | 0,04 | 0,23 | 0,02 | 0,10 | 0,01 | 0,06 |
|  |  | p = 0,7 | 0,00 | 0,19 | 0,00 | 0,06 | 0,00 | 0,03 | -0,03 | 0,14 | 0,01 | 0,05 | 0,00 | 0,02 | 0,01 | 0,12 | 0,00 | 0,04 | 0,00 | 0,02 |
|  | **5 Surveys / Year** | p = 0.1 | 0,65 | 1,09 | 0,20 | 0,38 | 0,02 | 0,12 | 0,57 | 1,04 | 0,23 | 0,40 | 0,05 | 0,14 | 0,39 | 0,71 | 0,10 | 0,25 | 0,00 | 0,10 |
|  |  | p = 0.3 | 0,08 | 0,37 | 0,05 | 0,17 | 0,02 | 0,07 | 0,10 | 0,38 | 0,05 | 0,17 | 0,03 | 0,08 | 0,11 | 0,33 | 0,05 | 0,15 | 0,03 | 0,09 |
|  |  | p = 0.5 | 0,01 | 0,20 | 0,01 | 0,07 | 0,01 | 0,04 | 0,01 | 0,16 | 0,01 | 0,07 | 0,01 | 0,04 | 0,02 | 0,14 | 0,01 | 0,07 | 0,01 | 0,04 |
|  |  | p = 0,7 | -0,01 | 0,17 | 0,00 | 0,05 | 0,00 | 0,02 | -0,01 | 0,14 | 0,00 | 0,04 | 0,00 | 0,02 | 0,00 | 0,11 | 0,00 | 0,03 | 0,00 | 0,02 |

Table 2. Mean and standard deviation of T-error for 144 simulated scenarios

|  |  |  | **Negative Trend** | | |  |  |  | **Stable** | |  |  |  |  | **Positive Trend** | | |  |  |  |
| --- | --- | --- | --- | --- | --- | --- | --- | --- | --- | --- | --- | --- | --- | --- | --- | --- | --- | --- | --- | --- |
|  |  |  | **N = 5** | | **N = 20** | | **N = 100** | | **N = 5** | | **N = 20** | | **N = 100** | | **N = 5** | | **N = 20** | | **N = 100** | |
|  |  |  | Mean | SD | Mean | SD | Mean | SD | Mean | SD | Mean | SD | Mean | SD | Mean | SD | Mean | SD | Mean | SD |
| **10 Years** | **3 Surveys / Year** | p = 0.1 | 0,02 | 2,07 | 0,04 | 0,73 | 0,01 | 0,29 | -0,01 | 0,11 | 0,00 | 0,05 | 0,00 | 0,02 | 0,02 | 0,77 | 0,02 | 0,38 | 0,00 | 0,17 |
|  |  | p = 0.3 | 0,05 | 0,95 | 0,01 | 0,44 | 0,00 | 0,19 | 0,00 | 0,07 | 0,00 | 0,03 | 0,00 | 0,02 | 0,00 | 0,50 | 0,02 | 0,24 | 0,00 | 0,11 |
|  |  | p = 0.5 | 0,01 | 0,79 | 0,01 | 0,37 | 0,00 | 0,17 | 0,00 | 0,06 | 0,00 | 0,03 | 0,00 | 0,01 | 0,00 | 0,41 | 0,00 | 0,22 | 0,00 | 0,10 |
|  |  | p = 0,7 | 0,05 | 0,72 | 0,03 | 0,35 | 0,00 | 0,16 | 0,00 | 0,05 | 0,00 | 0,03 | 0,00 | 0,01 | 0,01 | 0,42 | 0,01 | 0,21 | 0,00 | 0,09 |
|  | **5 Surveys / Year** | p = 0.1 | 0,04 | 1,24 | 0,04 | 0,56 | 0,02 | 0,25 | 0,01 | 0,09 | 0,00 | 0,04 | 0,00 | 0,02 | 0,04 | 0,67 | 0,00 | 0,32 | -0,01 | 0,14 |
|  |  | p = 0.3 | 0,04 | 0,79 | 0,01 | 0,40 | 0,00 | 0,18 | 0,00 | 0,06 | 0,00 | 0,03 | 0,00 | 0,01 | 0,00 | 0,46 | 0,00 | 0,24 | 0,00 | 0,10 |
|  |  | p = 0.5 | 0,05 | 0,75 | 0,02 | 0,36 | 0,01 | 0,16 | 0,00 | 0,05 | 0,00 | 0,03 | 0,00 | 0,01 | 0,00 | 0,40 | 0,00 | 0,21 | 0,00 | 0,09 |
|  |  | p = 0,7 | 0,02 | 0,70 | 0,01 | 0,34 | 0,00 | 0,16 | 0,00 | 0,05 | 0,00 | 0,03 | 0,00 | 0,01 | 0,01 | 0,38 | 0,00 | 0,19 | 0,00 | 0,09 |
| **20 Years** | **3 Surveys / Year** | p = 0.1 | 0,12 | 0,75 | 0,01 | 0,31 | 0,01 | 0,13 | 0,00 | 0,04 | 0,00 | 0,02 | 0,00 | 0,01 | 0,01 | 0,21 | 0,00 | 0,10 | 0,00 | 0,04 |
|  |  | p = 0.3 | 0,05 | 0,41 | 0,02 | 0,21 | 0,00 | 0,09 | 0,00 | 0,02 | 0,00 | 0,01 | 0,00 | 0,01 | 0,01 | 0,14 | 0,00 | 0,07 | 0,00 | 0,03 |
|  |  | p = 0.5 | 0,00 | 0,35 | 0,00 | 0,17 | 0,00 | 0,08 | 0,00 | 0,02 | 0,00 | 0,01 | 0,00 | 0,01 | 0,00 | 0,12 | 0,00 | 0,06 | 0,00 | 0,03 |
|  |  | p = 0,7 | 0,03 | 0,35 | 0,00 | 0,15 | 0,00 | 0,07 | 0,00 | 0,02 | 0,00 | 0,01 | 0,00 | 0,00 | 0,00 | 0,11 | 0,00 | 0,06 | 0,00 | 0,02 |
|  | **5 Surveys / Year** | p = 0.1 | 0,08 | 0,55 | 0,01 | 0,26 | 0,00 | 0,11 | 0,00 | 0,03 | 0,00 | 0,01 | 0,00 | 0,01 | 0,01 | 0,18 | 0,00 | 0,09 | 0,00 | 0,04 |
|  |  | p = 0.3 | 0,04 | 0,36 | 0,00 | 0,19 | 0,00 | 0,08 | 0,00 | 0,02 | 0,00 | 0,01 | 0,00 | 0,01 | 0,01 | 0,12 | 0,00 | 0,06 | 0,00 | 0,03 |
|  |  | p = 0.5 | 0,02 | 0,31 | 0,01 | 0,17 | 0,00 | 0,07 | 0,00 | 0,02 | 0,00 | 0,01 | 0,00 | 0,00 | 0,01 | 0,12 | 0,00 | 0,06 | 0,00 | 0,03 |
|  |  | p = 0,7 | 0,01 | 0,31 | 0,01 | 0,15 | 0,00 | 0,07 | 0,00 | 0,02 | 0,00 | 0,01 | 0,00 | 0,00 | 0,00 | 0,11 | 0,00 | 0,05 | 0,00 | 0,02 |
